# Wearable Sensors for Plants: Status and Prospects

**DOI:** 10.3390/bios15010053

**Published:** 2025-01-15

**Authors:** Xuexin Yan, Yawen Pang, Kaiwen Niu, Bowen Hu, Zhengbo Zhu, Zuojun Tan, Hongwei Lei

**Affiliations:** 1 College of Engineering, Huazhong Agricultural University, Wuhan 430070, China; xuexinyan123@webmail.hzau.edu.cn (X.Y.);; 2 College of Physics and Optoelectronic Engineering, Taiyuan University of Technology, Taiyuan 030024, China

**Keywords:** wearable sensors, plants, smart agriculture

## Abstract

The increasing demand for smart agriculture has led to the development of agricultural sensor technology. Wearable sensors show great potential for monitoring the physiological and surrounding environmental information for plants due to their high flexibility, biocompatibility, and scalability. However, wearable sensors for plants face several challenges that hinder their large-scale practical application. In this review, we summarize the current research status of wearable plant sensors by analyzing the classification, working principles, sensor materials, and structural design and discussing the multifunctional applications. More importantly, we comment on the challenges the wearable plant sensors face and provide our perspectives on further improving the sensitivity, reliability, and stability of wearable plant sensors for future smart agriculture.

## 1. Introduction

Smart agriculture [1,2] is a milestone in the development of agriculture and becomes an important symbol of agricultural modernization. As a modern agricultural production mode, smart agriculture improves agricultural production efficiency through real-time and accurate monitoring. However, the development of smart agriculture is severely limited by backward agricultural sensor equipment and detection technology. Sensors that can simultaneously and dynamically monitor the physiological information of plants and the surrounding environmental information are still lacking. In recent years, the rising and vigorous development of wearable technology has opened up new ideas for in situ real-time dynamic monitoring of plants. Wearable sensors have the advantages of good flexibility, good biocompatibility, and strong scalability, which is very promising for the long-term monitoring of plants. They can provide real and intuitive data feedback for plant management and help researchers understand the mechanisms of plant growth under different conditions such as plant stress [3,4] and plant diseases [5,6].

**Figure 1:**
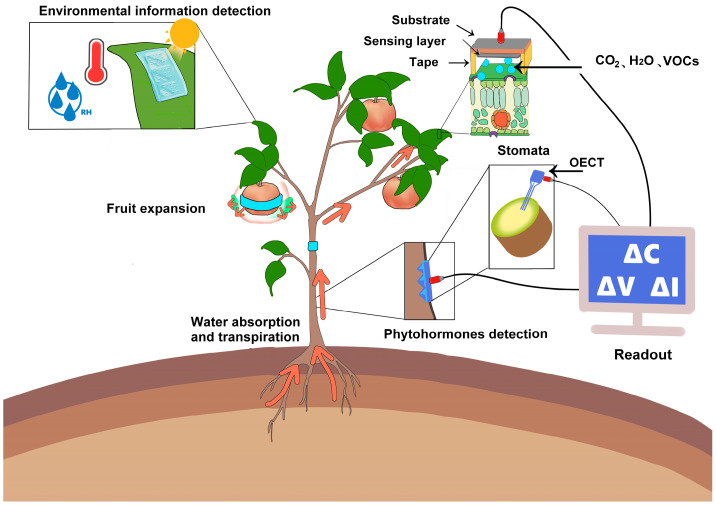
Schematic of the application of wearable plant sensors.

At present, a variety of flexible plant sensors with different functions have been developed, as shown in Figure 1. These sensors can be used to monitor the plant’s physiological information, such as water content [4,7,8], plant hormones [9,10], VOC (volatile organic compound) release [11,12], etc. They can also be used to monitor the microenvironment on the surface of plants, such as temperature and humidity, light [13,14], and harmful gases [15,16]. However, most of these sensors are not specifically designed for agriculture, and they can not survive under the real-world production of farming. Stable, low-cost, specific, and high-performance sensors for plants are still severely lacking.

The applications of these wearable sensors in the fields of plant physiological health monitoring, growth environment monitoring, etc., have been thoroughly explored in some review articles [17,18,19,20]. However, the importance of small molecules in plants, such as phytohormones and minerals, in the information transfer process should not be neglected, which will be analyzed in this paper. The material selection and preparation process of wearable plant sensors have been summarized in reviews [21,22], but the systematic compilation of their working principles and structural design is still insufficient. In view of this, this review will focus on the working mechanisms and structural design of wearable plant sensors, aiming to provide theoretical support and directional guidance for subsequent research and development. First, the classification and the working principles of wearable sensors for plants are introduced, and the material selection and structural design of wearable sensors are also summarized. Then, the application of wearable plant sensors in plant monitoring is elaborated from the aspects of plant physiological information monitoring and surrounding environment monitoring. Last, the existing challenges and problems in the research on wearable plant sensors are analyzed, and prospectives are proposed.

## 2. Research Status of Wearable Plant Sensors

### 2.1. Classification and Principles of Wearable Sensors

Flexible sensors can be classified into various types. Based on differences in sensing mechanisms, they are categorized as resistive sensors, capacitive sensors, piezoelectric sensors, and inductive sensors. Additionally, depending on their application, flexible sensors include strain and pressure sensors, gas sensors, humidity sensors, temperature sensors, and others. In the following sections, we will present a comprehensive overview of the working principles of flexible sensors, with an emphasis on their application-specific differences.

#### 2.1.1. Flexible Strain and Pressure Sensor

**Figure 2:**
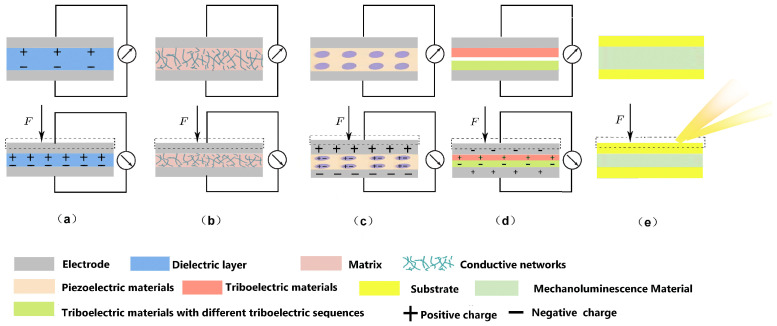
Flexible strain sensors with different sensing mechanisms: (**a**) capacitive; (**b**) piezoresistive; (**c**) piezoelectric; (**d**) triboelectric; (**e**) mechanoluminescence.

Flexible strain and pressure sensors exhibit high flexibility and stretchability. These sensors are highly sensitive to mechanical stimuli, enabling the conversion of mechanical deformation into an electrical signal output. From a sensing mechanism perspective, strain sensors and pressure sensors share fundamental similarities and can be broadly categorized into three types: capacitive, resistive, and piezoelectric (Figure 2 and Table 1). Despite these shared mechanisms, their measurement focuses and application scenarios differ. In the following discussion, we will use the flexible strain sensors, commonly employed to monitor plants, as an example to illustrate the working principles of these three types.

Capacitive flexible strain sensors typically consist of a parallel-plate capacitor structure, composed of flexible electrodes and dielectric layers. These sensors convert external mechanical signals into corresponding capacitive signals, with the capacitance calculated using the following formulate:

(1) $$C=\frac{\varepsilon_{0}\varepsilon_{r}A}{d},$$

where *ε*_0_ and *ε_r_* represents the vacuum permittivity and relative permittivity, *A* denotes the effective area of the electrode, and *d* is the spacing between the electrode plates. The capacitance changes in response to the external mechanical stress signal, primarily due to variations in the physical dimensions or the dielectric constant of the capacitor caused by external strain. The physical size of the sensor changes not only in the vertical direction, affecting the spacing between the plates, but also in the horizontal direction, altering the effective area of the plates (Figure 2a). Both changes contribute to variations in capacitance.

The working principle of a piezoresistive flexible strain sensor is to convert stress variations into changes in electrical resistance. This enables real-time monitoring of stress signals by observing variations in resistance values. Typically, these sensors comprise a flexible electrode, a conductive material, and a flexible substrate. It is important to note that the conductive materials must not only possess excellent electrical conductivity but also demonstrate sensitivity to stress changes, enabling deformation in response to external mechanical stimuli. The resistance *R* of a conductive material can be calculated as follows:

(2) $$R=\frac{\rho L}{S},$$

where *ρ* represents the resistivity, *L* denotes the length, and *S* is the cross-sectional area. As evident from the formula, changes in resistance primarily result from variations in the dimensions of the material caused by stress-induced deformation.

Changes in electrical resistance can be caused not only by changes in material dimensions, but also by changes in the spacing of the conductive fillers. In order to improve the conductivity of the material, one method is to incorporate the conductive filler as a second phase into a less-conductive matrix material. When pressure is applied to the sensor, the spacing of the conductive filler becomes smaller, and the concentration increases to the penetration threshold, forming a conductive network with a significantly reduced resistance, as shown in Figure 2b. A piezoelectric flexible strain sensor is a device that enables real-time monitoring of mechanical signals by measuring the voltage output of the sensor. Its sensing mechanism relies on the piezoelectric effect, which occurs in certain materials (e.g., crystals and ceramics) with non-centrosymmetric structures, allowing them to generate charges under mechanical stress. In the absence of external force, the charge centers of anions and cations in piezoelectric materials coincide, maintaining electrical neutrality. However, when subjected to an external force, the non-centrosymmetric structure of the crystal deforms, causing the charge centers of anions and cations to separate. This separation creates electric dipoles and leads to the generation of a piezoelectric potential (Figure 2c).

In addition to the stress detection methods mentioned above, the development of novel sensing mechanisms has significantly expanded the potential applications of flexible wearable electronic sensors. Notable examples include triboelectric flexible strain sensors, which utilize triboelectric and electrostatic sensing principles (Figure 2d), and flexible mechanoluminescence (ML) sensors that display dynamic forces in real-time (Figure 2e). However, triboelectric flexible strain sensors operate by converting mechanical movement into electrical energy and have limited applications in plant growth monitoring. Similarly, the luminescence changes of flexible force-emitting sensors are extremely short-lived and challenging to observe. Moreover, there is currently no widely accepted theory to fully explain the underlying mechanisms of this process [23]. In general, it is believed that ML originates from mechanically induced charge separation and/or bound charge displacement (polarization), which triggers various emission processes.

#### 2.1.2. Flexible Gas Sensors

Based on different detection principles, flexible gas sensors can be classified into several types, including semiconductor, electrochemical, optical, and surface acoustic wave sensors. Among these, semiconductor and electrochemical flexible gas sensors are particularly prominent in plant monitoring applications due to their excellent performance and applicability.

**Figure 3:**
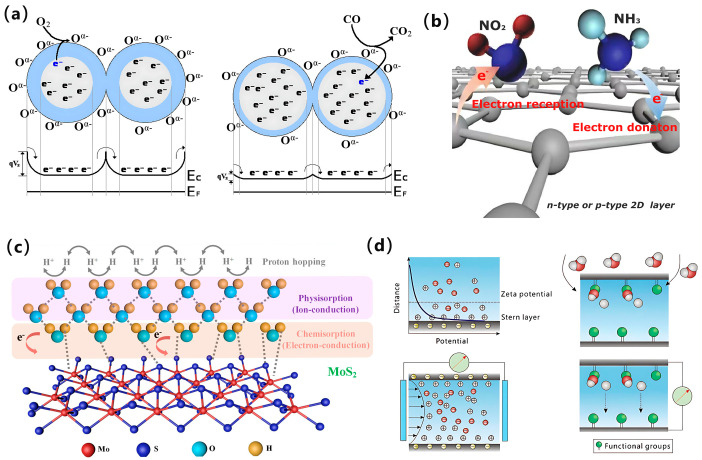
Schematic diagram of the sensing mechanism of the flexible gas/humidity sensor: (**a**) gas induction and resulting band bending in granular SMO films: oxygen adsorption to the surface, forming depletion zones; adsorbed oxygen reacts with CO, reducing the amount of band bending [28]. Copyright 2022, MDPI. (**b**) The charge transfer model explains the gas-sensing mechanism of two-dimensional layered materials [29]. Copyright 2018, Springer Nature. (**c**) Mechanism of MoS_2_ moisture sensor [30]. Copyright 2021, ACS. (**d**) Schematic mechanism for electrical generation based on streaming current and ion gradient diffusion [31]. Copyright 2020, Wiley.

Semiconductor-type flexible gas sensors can be categorized into resistive and non-resistive types based on the nature of their detection signals. Non-resistive gas sensors typically use Schottky diodes or field-effect transistors to measure changes in non-resistive parameters such as capacitance or potential barriers caused by gas interaction. However, the manufacturing process of this sensor is complex and unstable, resulting in limited research on this type. In contrast, resistive gas sensors, which have a more complex gas-sensing mechanism, are more widely studied. These sensors have two well-established theoretical models based on the type of gas-sensing materials: surface oxygen ion adsorption and charge transfer. The surface oxygen ion adsorption model is primarily used to explain the gas-sensing mechanism of metal oxide semiconductor gas sensors. For instance, in n-type metal oxides, the gas-sensing mechanism operates as follows (Figure 3a): in air, oxygen molecules are adsorbed onto the surface of the gas-sensitive material, reacting with electrons in the material to form oxygen anions. This process traps electrons, creating an electron depletion layer at the grain boundaries. This results in band bending and the formation of potential barriers, causing the n-type semiconductors to remain in a high-impedance state in the air. Conversely, when the sensor is exposed to a reducing gas such as CO, the CO molecules react with the oxygen anion to form CO_2_, releasing electrons back into the material. This increases the carrier concentration, reducing the electron depletion layer and placing the gas sensor in a low-impedance state. This resistance change enables the detection and quantification of specific gas concentrations in the environment.

On the other hand, the charge transfer model does not require an oxygen anion as an intermediary and is primarily used to explain the gas-sensing mechanism of two-dimensional materials, such as graphene (Figure 3b). In this model, gas molecules directly undergo charge transfer with the sensitive material, where the gain or loss of electrons depends on the conductivity type of both the gas being measured and the sensitive material. Typically, strong oxidizing gases (e.g., NO_2_) act as electron acceptors, removing electrons from the sensitive material and thereby increasing its resistance. Conversely, reducing gases (e.g., NH_3_) act as electron donors, supplying electrons to the sensitive material and decreasing its resistance. The resistance trend of the sensor should also be analyzed in conjunction with the conductive type of the sensitive material; exposure to oxidizing gases decreases the electron concentration, increasing the resistance. When exposed to reducing gases, the electron concentration increases, leading to a decrease in resistance. In contrast, p-type two-dimensional materials exhibit the opposite behavior: oxidizing gases increase their electron concentration, decreasing resistance, while reducing gases decrease the electron concentration, increasing resistance.

The sensing mechanism of the electrochemical flexible gas sensor relies on redox reactions between the gas and an electrolyte at the working surface. Gas diffusion induces a change in potential or current, enabling the determination of the gas composition and concentration. These sensors are commonly used to detect toxic and harmful gases, such as carbon monoxide (CO) and hydrogen sulfide (H_2_S).

#### 2.1.3. Flexible Humidity Sensors

A flexible humidity sensor consists of moisture-sensitive materials, flexible electrodes, and substrates. Among these components, the moisture-sensitive material serves as the core of the sensor and largely determines its performance. The sensing mechanism of most flexible humidity sensors is commonly explained by the Grotthuss chain reaction [24], which can be simplified as follows:

(3) $$H_{2}O+H_{3}O^{+}=H_{3}O^{+}+H_{2}O$$

Taking the MoS_2_ sensor as an example (Figure 3c), water molecules are chemisorbed onto the active sites (below 40% relative humidity), and electrons are withdrawn from the reduced water onto the p-type MoS_2_. As a result, the main carrier (hole) density decreases, the resistance increases, and the electron conduction mode dominates. After sufficient exposure to water (above 40% RH), the chemisorption layer begins to physisorb while a continuous water layer is formed. The water molecules are ionized to H_3_O^+^ by the electrostatic field and are transferred according to the Grotthuss chain reaction. Based on the electrical signal output, flexible humidity sensors are typically categorized into resistive, capacitive, impedance, and voltage types. Among these, resistive flexible humidity sensors utilize moisture-sensitive materials to alter resistance through the adsorption and desorption of water molecules. Depending on the type of charge carriers, resistive humidity sensors can be further classified into two categories: electronically conductive and ionic. In contrast to electron-conductive types, where the carriers are free electrons, ionic conductive currents are generated by the ionization of a liquid electrolyte within a moisture-sensitive material in an aqueous solution or molten state, enabling the movement of anions and cations. For instance, the electrical response strength of a humidity sensor based on a dual-network structure of organic hydrogels [25] is primarily influenced by the mobility and concentration of charge carriers (e.g., K^+^ and Cl^−^). Capacitive humidity sensors, on the other hand, operate by altering the dielectric constant of the material through the adsorption or desorption of water molecules, which impacts the capacitance value and produces distinct humidity response signals.

Impedance-based flexible humidity sensors determine impedance by measuring the current response to a sinusoidal voltage applied to the sensor in the frequency domain. Generally, the impedance output of such sensors strongly correlates with relative humidity (RH) and frequency. Specifically, impedance typically decreases as RH and frequency increase. At higher frequencies, the impedance curve becomes flat, indicating that the polarization of water molecules cannot keep pace with the rapid change in the electric field. Under these conditions, impedance becomes independent of RH. Therefore, it is crucial to study the optimal operating frequency of the flexible humidity sensor before conducting actual humidity monitoring.

Unlike traditional flexible humidity sensors that require an external power supply, self-powered voltage-based flexible humidity sensors have garnered significant attention. These sensors generate electricity when moisture interacts with moisture-sensitive materials [26,27]. Currently, two primary theories explain this phenomenon: flow currents and ion drift caused by concentration gradients, as shown in Figure 3d. The voltage or current output is strongly correlated with changes in relative humidity around the device, enabling flexible self-powered operation without the need for an additional power supply. This voltage-based flexible humidity sensor offers a promising approach for developing self-powered and environmentally friendly sensing systems, contributing to a sustainable future.

#### 2.1.4. Flexible Temperature Sensors

At present, temperature measuring methods are primarily categorized into two types: non-contact and contact, with flexible temperature sensors falling under the contact temperature category. Based on their sensing mechanisms, flexible temperature sensors can be mainly classified into flexible resistance temperature detectors (RTDs), flexible thermistors, and flexible thermocouples (Table 2). Flexible resistance temperature detectors primarily rely on the intrinsic properties of a heat-sensitive material to detect temperature. Materials such as gold, platinum, nickel, silver, and copper exhibit changes in resistance as temperature varies. The relationship between resistance and temperature is described by the following equation [32]:

(4) $$\frac{\Delta R}{R_{0}}=\alpha \left(T-T_{0}\right),$$

where *R*_0_ represents the initial resistance at temperature *T*_0_, and *α* stands for the temperature coefficient of resistance. As indicated by the equation, the change in resistance of a flexible resistance temperature detector is directly proportional and linear to the change in temperature.

**Figure 4:**
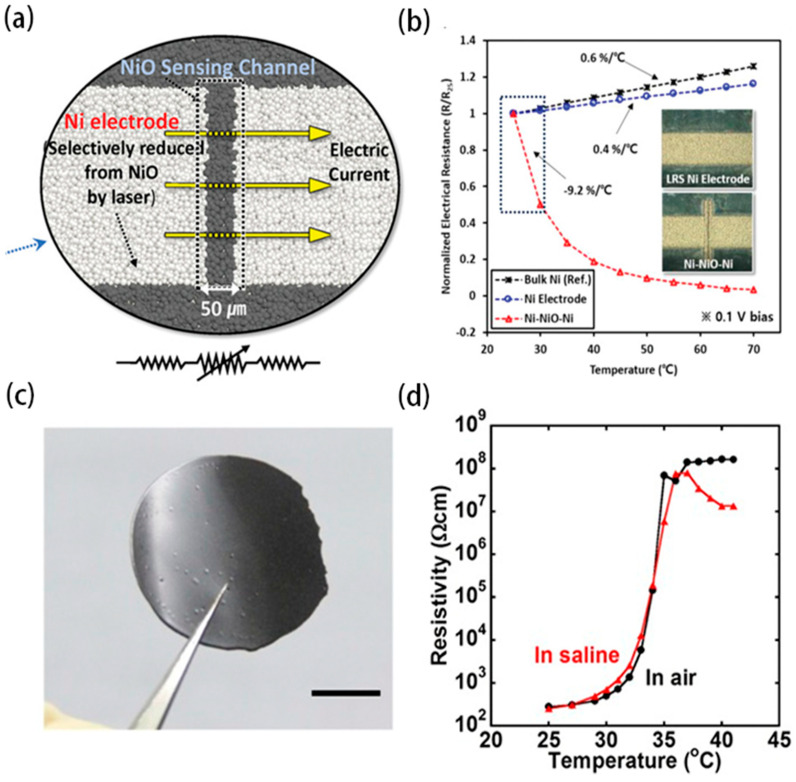
(**a**) Negative temperature coefficient thermistor based on nickel oxide [33]. Copyright 2020, ACS. (**b**) Negative temperature coefficient thermistor response to temperature [33]. Copyright 2020, ACS. (**c**) Positive temperature coefficient thermistor based on graphene particles [34]. Copyright 2015, PNAS. (**d**) Positive temperature coefficient thermistor response to temperature [34]. Copyright 2015, PNAS.

Unlike flexible resistance temperature detectors, flexible thermistors exhibit nonlinearity due to the intrinsic properties of their thermally sensitive materials. Flexible thermistors are generally categorized into positive temperature coefficient thermistors (PTCs) and negative temperature coefficient thermistors (NTCs). Figure 4a demonstrates a schematic of the structure of a nickel oxide NTC-based thermistor, while Figure 4b shows the response curves of this type of thermistor to temperature changes. Correspondingly, Figure 4c,d show the PTC thermistor based on graphene particle, and its response characteristics to temperature changes. PTC thermistors are typically composed of ceramic or polymer composites. In polymer-based thermistors, the concentration of conductive filler lies near the percolation threshold. Although the fillers do not make direct contact, they transfer charge via the tunneling effect.

Below the percolation threshold, the resistance is extremely high; once the threshold is exceeded, the conductivity increases rapidly. The resistance of polymer composites is primarily determined by the spacing of the fillers. As temperature increases, the polymer substrate expands, increasing the filler spacing, weakening the tunneling effect, and causing the resistance value to rise. In contrast, NTC thermistors are made from semiconductor materials or metal oxides, such as nickel oxide or iron oxide. At high temperatures, carrier transitions and thermal excitation increase the number of charge carriers, thereby reducing the resistance of the NTC thermistor.

In general, the relationship between a thermistor’s resistance and the temperature can be described by the following formula:

(5) $$R_{T}=R_{\infty }e^{\left(\frac{B}{T}\right)},$$

where *R_T_* represents the resistance value at temperature *T*, *B* is the thermal constant, and *R*_∞_ denotes the reference resistance. Although the response of the flexible thermistor to temperature is nonlinear, it can be approximated as linear within a small temperature range, similar to the response of a corresponding flexible resistance temperature detector.

Flexible thermocouples operate based on the Seebeck effect to generate an electromotive force. Two heat-sensitive materials are used to form a closed loop, creating a hot end and a cold end. The hot end is connected to the object being measured, while the cold end is attached to the object with a known temperature. The temperature difference between the hot and cold ends causes charges within the heat-sensitive material to move in a specific direction, resulting in an electric current. In addition to electrical methods, temperature detection can also be achieved through optical means. A flexible thermochromic sensor is a visual temperature sensor that changes the color of a substance in response to temperature variations. Reversible thermochromic materials return to their original color after the temperature changes, while irreversible thermochromic materials undergo a permanent color change upon reaching a certain temperature. The latter is commonly used to indicate temperature limits or changes for safety warnings. Thermochromic materials such as metal complexes [35,36], gold and silver nanoparticles [37,38], and liquid crystals [39,40,41] have garnered significant attention for their potential applications in the field of human–computer interaction, owing to their ability to provide direct visual feedback.

### 2.2. Material Selection for the Flexible Sensors

The materials used in flexible sensors can be categorized into four types: substrate materials, packaging materials, functional materials, and electrode materials. To integrate with the leaves, stems, roots, and other plant organs while monitoring physiological and environmental information, flexible sensors must possess mechanical properties similar to those of plant tissues. Consequently, the materials used in these flexible sensors typically exhibit ductility and flexibility. They are often composed of natural flexible materials or rigid materials in the form of thin films. Table 3 summarizes and compares the various materials required for the preparation of plant flexible sensors

#### 2.2.1. Substrate Materials and Packaging Materials

Plants undergo deformation during growth and development, and to achieve long-term in situ monitoring of organ physiology and environmental information, the substrate and packaging materials used for flexible sensors usually have a low elastic modulus and extensibility and flexibility and can undergo deformation along with the organ. At the same time, for the normal growth and development of plants, without affecting the normal photosynthesis and gas exchange, it should also require good biocompatibility, light transmittance, and air permeability.

Common substrate and packaging materials include polydimethylsiloxane (PDMS), copolyester (Eco-flex), polyurethane (PU), aerogel, hydrogel, polyethylene terephthalate (PET), polyurethane, etc. Among these, PDMS, a natural flexible material, has an elastic modulus ranging from 1 to 150 MPa after curing and a maximum elongation rate of up to 300%. Due to its low elastic modulus and notable extensibility, PDMS is widely used as a flexible substrate and packaging material for plant wearable sensors. In addition, PDMS exhibits excellent light transmittance and air permeability. Eco-flex, known for its superior stretchability, is frequently used to fabricate plant flexible sensors requiring significant deformation. Zhang et al. [42] leveraged the difference in elastic modulus between PDMS and Ecoflex to transfer laser-induced graphene (LIG) patterns onto Ecoflex, forming a sandwich-structured AWS sensor. This structure, with LIG embedded between the Ecoflex layers, enables real-time monitoring of tomato plant growth and water status by measuring plant pulse. Polyurethane (PU) is a polymer material composed of a flexible soft segment formed by polyols and a rigid hard segment formed by the basic urethane group (NHCOO). This unique microphase structure imparts polyurethane with excellent elasticity and strength, as well as remarkable tensile and tear strength. Liang et al. [43] developed polyurethane wet-sensitive fibers by incorporating graphene (Gr) into the surface of molten PU fiber as a substrate. When the graphene content reached 4.1%, the fracture strength of the wet-sensitive fiber achieved 94.9 MPa, and the fracture elongation reached 91.5%, demonstrating the superior tensile and tear strength of PU.

As an emerging flexible electronic material, aerogel consists of interconnected three-dimensional nanosolid networks and air-filled pores. This 3D porous structure gives the aerogel excellent light and air permeability, which ensures smooth gas exchange and photosynthesis in plants. In addition, the inherent flexibility of the aerogel and the conductive network formed by its compressed 3D porous structure enable aerogel-based flexible pressure sensors to exhibit high sensitivity, rapid response, and excellent recovery capabilities. Yang et al. [44] successfully developed a pressure sensor based on a spider-web-inspired (PIF/CNT) conductive composite aerogel, achieving a wide linear sensing range of 0.015–3.34 kPa, an ultra-low detection limit of 10 Pa, and a high sensitivity of 0.507 kPa^−1^. Furthermore, the aerogel demonstrated a fast response and recovery time of 85 ms and 80 ms, respectively, along with a rapid compression response speed of 500 mm min^−1^. Hydrogels, on the other hand, are highly absorbent polymers capable of altering 3D cross-linking structures to regulate water content and film properties. With external changes such as pressure, bending, or temperature, the flexibility and plasticity of hydrogels enable changes in their shape or volume, facilitating the perception and measurement of physical quantities and environmental parameters. Hsu et al. [45] synthesized a polyacrylic acid (PAA) hydrogel with a dual-network structure, combining it with a graphene and polyaniline coating to monitor the growth of aloe leaves and pepper plants for up to 18 days. Polyethylene terephthalate (PET) is a widely used thermoplastic polymer valued as an encapsulation material for its excellent electrical insulation properties, mechanical strength, and viscosity. Compared to PET, polyimide (PI) exhibits superior heat resistance and exceptional environmental stability. Leveraging these properties, Im et al. [3] developed flexible plant sensors based on PI to monitor drought stress on the surface of crop leaves. To enhance the mechanical stress tolerance and adhesion of the sensor, the PI film was laminated with the single-side adhesive PET film as a substrate, allowing it to adhere securely to the back of the leaf. This design effectively mitigated external force damage caused by clamping.

#### 2.2.2. Functional Materials

As the core component of flexible sensors, functional materials play a critical role in enhancing sensor sensitivity. Generally, functional materials are categorized into conductive materials and various semiconductor materials. Common conductive materials used in flexible sensors include metals, conductive polymers such as polypyrrole and polyaniline, and carbon-based nanomaterials such as carbon fibers, carbon nanotubes, and graphene.

Metal materials, such as metal nanowires (including silver, gold, and copper nanowires) and metal nanoparticles, are widely utilized to enhance the electrical performance of sensors due to their exceptional electrical conductivity. Among these, silver nanowires are considered an outstanding material for flexible sensors, offering excellent electrical conductivity, high light transmittance, and superior bending resistance. Zhu et al. [46] successfully developed a conductive hydrogel-based flexible sensor by printing permeable silver nanowires onto a tough supramolecular hydrogel. The resulting flexible strain sensor demonstrated excellent electrical performance and mechanical properties and has a notable self-healing capability.

Liquid metal is an ideal material for fabricating large-deformation flexible sensors due to its low melting point, high electrical conductivity, unlimited deformability, and excellent compatibility. Flexible sensors based on liquid metal have garnered significant attention, demonstrating exceptional performance in strain, pressure, and bending applications, as well as stability under complex deformation conditions. Qu et al. [47] successfully developed a liquid metal-based plant electron tattoo, which exhibited high stability and sensitivity under complex deformation. This innovation is particularly suitable for the continuous monitoring of the water content and electrical signals of plants, offering valuable insights into the mechanism of drought adaptation and facilitating the development of drought-resistant crop varieties. Conductive polymers achieve conductivity through doping with oxidants, reductants, or protic acids, exhibiting electronic and optical properties similar to those of metals. These properties make conductive polymers suitable for the development of wearable sensors for plants. Conductive polymers are generally categorized into two types: natural conductive polymers and composite conductive polymers.

Common natural conductive polymers include polypyrrole (PPy), polyaniline (PANI), and polyethylene dithiophene (PEDOT). Leveraging the high conductivity and flexibility of PEDOT, MEDER et al. [48] has successfully developed compliant, self-adhesive electrodes based on PEDOT:PSS. These electrodes adhere to plant surfaces solely through van der Waals forces, enabling non-invasive surface potential recording while avoiding potential damage from electrode insertion or ion diffusion. Additionally, these self-adhesive electrodes, combined with a stretchable silver conductor paste, can monitor plant electrical signals, providing a new tool for plant scientific research.

Carbon-based materials, such as carbon fiber, carbon nanotubes (CNTs), and graphene, are extensively used in the preparation of plant flexible sensors due to their high conductivity, flexibility, and stability. Carbon fiber is renowned for its light weight, high strength, and chemical stability, with a typical diameter ranging between 5 and 10 microns. These properties make it an important material for flexible sensor development. Owing to the continuity and integrity of carbon fibers, they are frequently employed in the manufacturing of flexible strain sensors. However, these sensors often exhibit poor sensitivity under small strain conditions [49,50]. As a one-dimensional (1D) nanomaterial, CNTs possess exceptional tensile strength, high conductivity, and controllable micropore size. Additionally, the chemical stability of the CNT solution enables direct deposition onto flexible substrates via solution treatment technologies, forming continuous conductive networks that remain intact even under significant tensile deformation. Amjadi et al. [51] developed an Ecoflex-CNT-Ecoflex sandwich-structured flexible strain sensor using CNTs. By controlling the density of the CNT permeation network, the sensor achieved high sensitivity and a wide strain detection range exceeding 1380%.

Graphene, a two-dimensional (2D) carbon-based material, has garnered significant attention due to its high transmittance, large specific surface area, high electrical conductivity, and high elasticity. Its super permeability to water molecules demonstrates significant potential for flexible humidity sensors. Zeng et al. [52] implemented a self-powered humidity sensor using a flexible graphene oxide (GO) membrane, achieving an ultra-fast response and recovery time of less than 0.3 s. Moreover, the sensor exhibited high sensitivity across a wide relative humidity (RH) range of 33% to 98%, enabling the monitoring of surface humidity in gardenia leaves for up to 60 h.

#### 2.2.3. Electrode Material

Electrodes are integral to flexible sensors, functioning similarly to the blood vessels and nerves of living organisms. They must be thin, highly tensile, and flexible while retaining excellent electrical conductivity.

Traditional rigid sensors typically use metals such as silver and copper as electrode materials. To accommodate the requirements of flexible sensors, these metal electrodes can be designed into stretchable structures, such as folds and ripples, to enhance flexibility. While these structures offer excellent electrical conductivity, their fabrication often relies on complex microprocessing techniques, including lithography, electron beam deposition, and reaction ion etching. These methods are expensive and unsuitable for large-scale applications. An alternative is to use liquid metal, which combines excellent electrical conductivity with extremely high flexibility, characterized by a near-zero Young’s modulus. However, due to its inherent mobility, liquid metal requires compound processing or encapsulation before use. In addition to metals, carbon nanotubes and graphene have emerged as excellent electrode materials due to their high electrical conductivity and flexibility. However, using these materials alone may not yield optimal performance. As a result, current research primarily focuses on composite materials that integrate conductive components, such as carbon nanotubes, graphene or metal nanowires, and metal nanoparticles, with flexible substrate materials like PDMS, PU, or PI. This combination not only offers a diverse range of material choices but also facilitates easy integration, making it one of the most promising approaches for the preparation of flexible electrode materials.

**Table 3:** Material selection of flexible sensors.

Materials	Characteristics	Application Scenarios
DMS	Flexibility, scalability, transmittance, gas permeability, hydrophobicity, and adhesion	Flexible substrate, packaging
Eco-flex	Flexibility, scalability, transmittance, gas permeability, adhesion, and degradability	Flexible substrate
PU	Flexibility, scalability, transmittance, gas permeability, adhesion, and anti-biological aging	Flexible substrate
Aquogel	Flexibility, compressibility, high sensitivity, low cost, transmittance, and gas permeability	Flexible substrate
Aerogel	Flexibility, compressible, high sensitivity, and transmittance	Flexible substrate
PET	Electrical insulation, strong mechanical properties, transmittance, and printability	Flexible substrate and packaging
PI	Heat resistance, environmental stability, scalability, and transmittance	Flexible substrate and surface packaging
Silver nanowire	Conductivity, stability, transmittance, flexure resistance, and scalability	Functional materials and flexible electrodes
Liquid metal	Low melting point, high conductivity, infinite deformation, good compatibility, and low viscosity	Functional materials and flexible electrodes
Conductive polymer	Combines easily with substrates and electrical conductivity	Function material
Carbon-based materials	Transmittance, large specific surface area, high electrical conductivity, elasticity, and thermal conductivity	Functional materials and flexible electrodes

### 2.3. New Structural Design of Flexible Sensor

To enhance the sensing performance of wearable plant sensors, both the selection of flexible materials and the structural design of sensors play a critical role. Specific structural designs can enable rigid materials with a high Young’s modulus to achieve varying degrees of stretchability while maintaining relative stability under multi-modal deformation.

**Figure 5:**
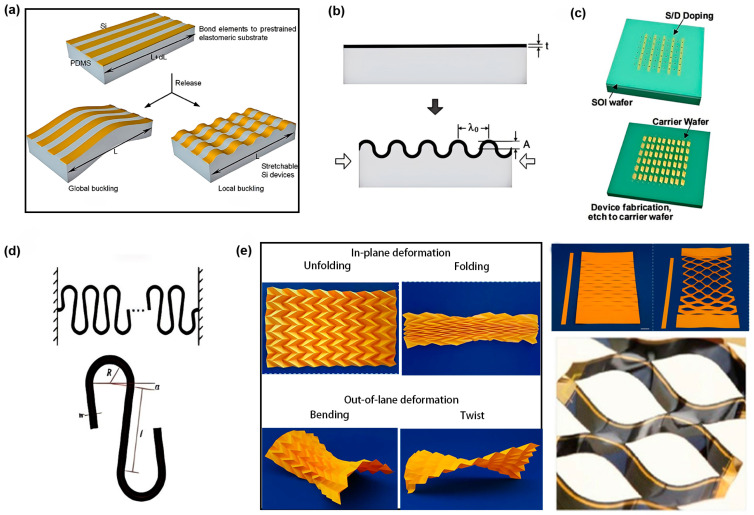
(**a**) Schematic diagram of the formation of the wave structures [56]. Copyright 2008, AIP. (**b**) The wavelength and amplitude of the wave structure and the substrate prestrain [57]. Copyright 2010, Wiley. (**c**) The formation process of the island–bridge structure [58]. Copyright 2009, Wiley. (**d**) Serpentine structure formation [59]. Copyright 2018, Elsevier. (**e**) Origami and paper-cut designs [60,61]. Copyright 2010, Elsevier.

Inspired by the folds of skin and creases in fabric, researchers have designed wave-like structures to enhance the scalability of inorganic materials. As illustrated in Figure 5a, when a flexible substrate is released through thermal expansion, solvent swelling, or mechanical pre-stretching, the inorganic film on the substrate’s surface undergoes localized buckling to form a wave or wrinkle pattern. This design converts the substrate’s compressive tensile strain into bending strain within the film, effectively preventing fracture caused by local buckling. Localized buckling of the film is accompanied by global buckling, which contributes minimally to the enhancement of tensile properties. The distinction between these two modes depends on the modulus and thickness ratio between the flexible substrate and the inorganic films. For instance, in the case of PDMS and silicon (Si), a thickness ratio greater than 1000 promotes the formation of localized buckling patterns, while a ratio below 1000 facilitates global buckling patterns.

When a tensile or compressive strain occurs, the amplitude and wavelength of the wave or wrinkle profile change accordingly (Figure 5b). This change is determined by the mechanical properties and geometric characteristics of the inorganic films and flexible substrates. Numerous mechanical models have been developed to predict wave or wrinkle configuration and their corresponding tensile properties [53]. During the growth cycle, certain plant organs may undergo deformations several times larger than their original size. Thus, plant wearable sensors must not only accommodate bending but also exhibit high ductility to enable long-term monitoring throughout the entire growth cycle. However, the wave configuration alone achieves only about 20% ductility along the ripple direction. To address this limitation, researchers have designed flexible wearable devices based on the island–bridge structure [54]. In this design, the ‘island’ contains sensing units and circuit components, while the “bridge”, composed of polymer and metal, is freely suspended between the island as an interconnection. When the island–bridge structure is transferred onto a prestrained flexible substrate, the island adheres fully to the substrate, while the bridge arches under van der Waals force, forming an arc when the substrate releases the prestrain. During deformation, the bridge absorbs most of the strain, ensuring that the functional island experiences minimal deformation (Figure 5c).

Planar scalable structure designs are also crucial for the development of flexible sensors in plants. These designs often incorporate reproducible microstructures, such as serpentine film-like networks, which use unique curved layouts to significantly enhance the stretchability of rigid materials. By reducing strain during stretching, these structures improve the mechanical performance of sensors. To further optimize tensile and mechanical properties, researchers have introduced fractal designs, developing self-similar serpentine structures (Figure 5d). These structures achieve greater malleability and functional density by increasing the self-similarity order and filling rate. Each addition to the self-similar structure increases the extension rate of the serpentine structure by at least twofold. This ensures that the sensors can securely attach to the plant organs, enabling monitoring throughout the growth cycle and providing comprehensive plant growth data.

Origami and paper-cut structures also offer unique value in flexible sensor design (Figure 5e). Origami structures enable flexible bending and stretching through predefined creases. The maximum principal strain on the parallelogram surface is nearly zero, resulting in low stiffness upon stretching. While paper-cut structures share similarities with origami, their key distinction lies in achieving flexibility through cutting, allowing for three-dimensional deformation and high stretchability. For certain stretchable electronic devices, paper-cut design can achieve ultra-high stretchability. With specific patterned defect configurations, the stretchability can reach up to 370% [55]. The application of these traditional techniques in modern engineering provides new avenues for innovation in flexible electronic devices.

Structural design can also draw inspiration from bionics to develop flexible sensor architectures that enable novel sensing mechanisms or enhance specific physical functions, such as adhesion. For example, Zhang et al. [42] created an integrated flexible plant system based on an adaptive wound strain sensor. This sensor wraps directly around the stem of a tomato plant without the need for adhesives, enabling real-time monitoring of growth and water status in tomato plants.

## 3. Applications of Wearable Plant Sensors

### 3.1. Detection of Plant Physiological Information

#### 3.1.1. Water Content Detection

The water content in plants is the main part of the protoplasm and represents the key physiological information of plants [62]. Usually, the water content is about 80–90%. Monitoring and controlling the water content in plants can not only avoid adverse reactions after drought stress and safeguard their normal physiological activities but also improve the efficiency of accurate irrigation in agriculture and promote the development of water-saving agriculture [63].

**Figure 6:**
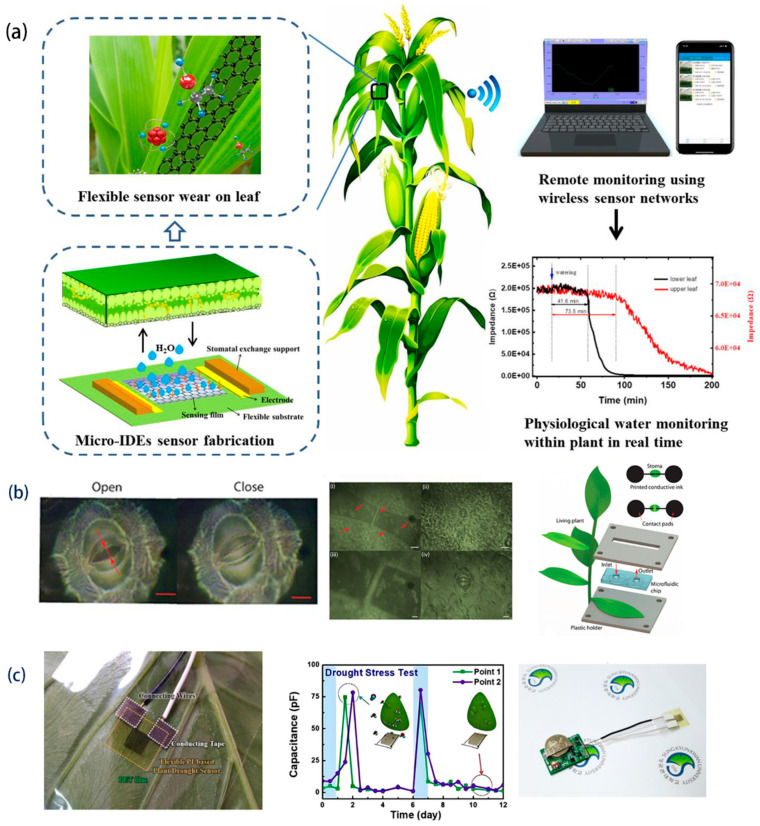
Example of water content detection application. (**a**) Dynamic monitoring of water transport within plants [8]. Copyright 2022, MDPI. (**b**) Nondestructive monitoring of plant moisture status using conductive ink based on CNTs [4]. The first figure shows microscope pictures of a stoma in the opened and closed states with the stomatal aperture indicated. The second figure presents bright-field microscopy images of a microfluidic chip aligned on top of a single stoma (i, iii) and the same stoma after printing (ii, iv). Red arrows point to individual stomata. The third figure illustrates schematics of conductive circuits printing on the leaf surface. A microfluidic chip is placed on top of the leaf abaxial surface and clamped in between two holders. Copyright 2017, Royal Society of Chemistry. (**c**) Real image of the flexible PI-based plant drought sensor attached on the lower surface of leaves and Nicotiana tabacum responses to drought stress over time [3]. Copyright 2018, MDPI.

Plants absorb water through roots, transfer it to leaves via stems, and ultimately release it into the atmosphere through transpiration [64]. Within this cyclic process of water absorption, transport, and dissipation, the movement of water from the stem to the leaves is vital. Sensors play an important role in understanding the transport process of water in plants. For example, Li et al. [8] designed a moisture sensor based on flexible nano-graphene oxide (GO). This sensor had high sensitivity, fast response, and good biocompatibility. By installing the sensors on different leaves, the water transport time from the root to each tested leaf was quantified, and dynamic monitoring of water transport within the plant was realized. (Figure 6a). However, the energy supply of such sensors becomes a challenge due to the low conductivity and high-power consumption of graphene oxide. Meanwhile, Chai et al. [7] employed the principle of spatial anisotropy of heat transfer during stem flow, utilizing a flexible PTC (positive temperature coefficient) thermistor to achieve continuous monitoring of plant sap flow in complex environments. Furthermore, plants have different diameters of stalks under different moisture conditions [65]. Therefore, monitoring changes in stalk diameter can determine the moisture status of the plant. Zhang [42] et al. developed an integrated plant flexibility system based on strain sensors, which realized the real-time monitoring of stalk diameter and thereby reflected the moisture condition in tomato plants.

Besides water transportation within the stalk, the process of transpiration and dissipation from the leaves is also important in the plant water cycle. Stomata, as the gateway for gas exchange with the external environment, have a big effect on water transpiration [66]. Koman et al. [4] prepared a conductive ink based on CNTs (carbon nanotubes), and the ink was then directly printed on both ends of the stomata to form a circuit that is responsive to the opening and closing states of the stomata. When the stomata are closed, the CNT ink makes contact, and the circuit is connected to form a current, while when the stomata are open, the circuit is disconnected, and the current disappears. (Figure 6b) This approach provides an innovative technological means for nondestructive monitoring of plant water status.

In addition, the transpiration and opening and closing of stomata are affected by the environmental humidity. The vapor pressure deficit (VPD) is defined as the difference between the water vapor pressure and the saturated water vapor pressure in the air [67]. Transpiration occurs as long as VPD exists in the atmosphere and the transpiration rate decreases as the VPD decreases. Vurro et al. [68] designed an invasive sensor based on organic electrochemical transistors to monitor the VPD of plants and determine the water content of the plants through resistance change. Considering that the use of invasive sensors may cause damage to plants, noninvasive sensors are gaining more attention. Atherton et al. [69] first proposed a leaf surface humidity sensor, which consisted of a thin-film micro-heater and two thin-film thermocouples. The thermocouples were used to record the temperature difference induced by the heater, thus assessing the moisture content of the leaf. Although this sensor demonstrated initial flexibility by using flexible PI film, it still needed to be fixed to the leaf surface with the help of a plastic fixture, which could cause irreversible damage to the plant in the long term. Im et al. [3] prepared a flexible sensor by transferring a PI film with Ti/Au electrodes onto a unilateral adhesive PET film. This structure can realize the complete flexibility of the sensor armature and integrate the sensor with the plant without the need for additional rigid structures. This sensor is useful for drought stress response studies in botany, for example, with tobacco, by monitoring capacitance changes to obtain real-time water status (Figure 6c). Since the modulus of elasticity of PI is much larger than that of the leaf surface, it imposes some physical limitations on leaf growth and is not suitable for long-term monitoring. Moreover, PET as a support layer may lead to water vapor accumulation between the sensor and the leaf surface, which would also affect the sensor stability. Kim et al. [5] prepared chlorine-doped PEDOT electrodes on the surface of plant organs by vapor-phase printing, which enabled the monitoring of damage caused by dehydration and UV exposure during the plant life cycle. Despite advances in this technology in the laboratory, the preparation process requires the placement of leaves in a vacuum reaction chamber for vapor phase deposition, making it difficult to scale up in real-world agricultural scenarios.

#### 3.1.2. Mineral Element Detection

Mineral elements play a key role in plant survival and development. The essential mineral elements such as carbon, hydrogen, and oxygen for plants come from different sources. Glucose as an example, is a key substrate for plant cellular respiration and also a source of energy for various plant life activities. Therefore, real-time monitoring of glucose can provide crucial data supporting healthy plant growth, development, and reproduction. Wearable devices showed great potential in the real-time monitoring of mineral ion uptake and transportation and mastering the content of mineral ions in plants.

**Figure 7:**
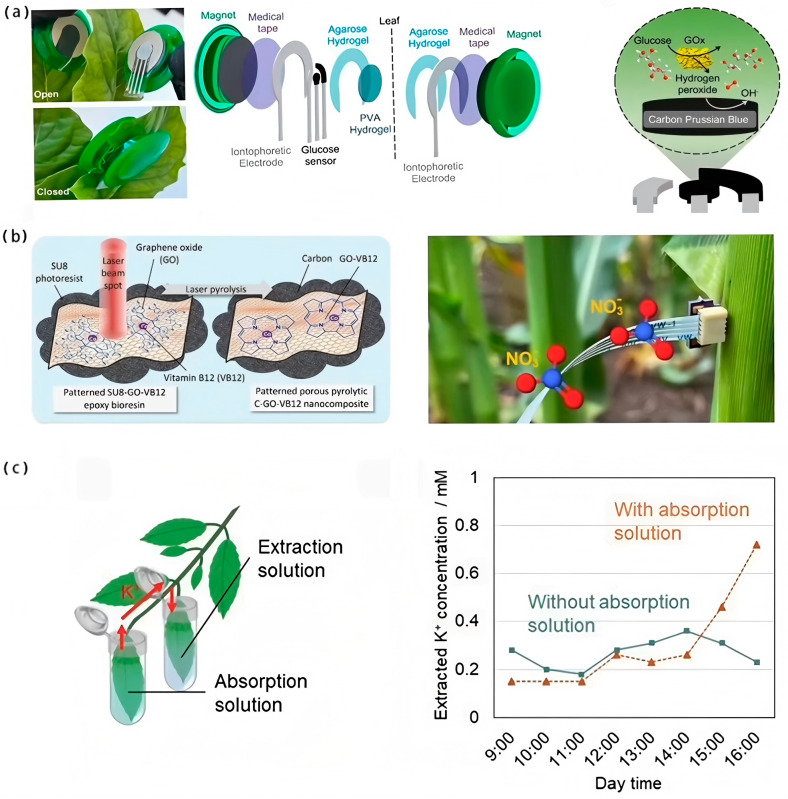
Example of mineral element detection application. (**a**) Glucose-selective sensor combined with reverse iontophoresis [70]. Copyright 2023, Elsevier. (**b**) Plant nitrate sensor based on a photosensitive epoxy resin [76]. Copyright 2022, ACS. (**c**) Invasive extraction and continuous monitoring of K+ in plant leaves using the leaching method [74]. Copyright 2023, Elsevier.

Perdomo et al. [70] combined a glucose-selective sensor with reverse iontophoresis by applying a current between an anode and a cathode so that glucose flowed to the measuring electrode (cathode) in less than 10 min. In the presence of the glucose oxidase-modified working electrode of the sensor, oxygen reacts to produce hydrogen peroxide (H_2_O_2_). Quantitative detection of glucose is achieved by detecting the amount of H_2_O_2_ produced or the amount of oxygen consumed (Figure 7a). Note that this process does not cause any damage to the plant leaves. Another effective method for the detection of glucose is using invasive wearable sensors. To minimize the damage caused by the penetration of the sensors into the plant, sensors have evolved from traditional large electrodes to microelectrodes. For instance, Diacci et al. [71] successfully applied a minimally invasive organic electrochemical transistor sensor (OECT) on the phloem of plant stems to monitor the concentration of metabolites and, therefore, quantify the glucose content in the plant. Nevertheless, the wounds caused by this invasive assay, even if minimal, may affect the reliability of the plant sensor’s long-term measurements during the healing process. Meanwhile, how to prevent enzyme detachment during electrode insertion into plant tissues is a topic that deserves further discussion, and hydrogel-based protected microneedle electrodes provide a new strategy to solve this problem [72,73].

In addition to carbon, hydrogen, and oxygen, there are numerous soil-derived mineral elements. Among them, nitrogen is the basis of proteins and is involved in many physiological processes in plants; potassium enhances plant resistance and regulates the cellular osmotic pressure. Real-time monitoring of the levels of these elements, such as nitrogen and potassium, is essential for precise regulation of plant growth and yield improvement. Nagamine et al. [74] performed the first minimally invasive extraction and continuous monitoring of K^+^ in plant leaves using the leaching method. A wearable K+ sensor membrane was used to extract K+ from tobacco leaves in a minimally invasive manner by soaking the leaf in a phosphate-buffered saline (PBS) extraction solution (Figure 7c). However, it should be noted that the extraction solution evaporates gradually during the measurement, which limits the sensor’s capacity for long-term monitoring. Also, it is not clear whether the concentration of the extracted compounds can quantitatively represent their chemical composition in the leaf.

OECT sensors are widely used in the field of ion detection through the doping/de-doping of channel polymers by ions in the electrolyte triggered by changes in gate voltage. Strand et al. [75] doped polyvinyl chloride (PVC)-based ion-selective membranes (ISMs) between the electrolyte and the PEDOT:PSS channels and succeeded in realizing highly sensitive K+ detection and selective sensing. However, ISM hinders the ion transport efficiency to some extent, which reduces the grating response. To solve this problem, sorbitol additives were added into the PEDOT:PSS inks, which was demonstrated to improve the gate response and sensitivity of the devices. In addition to ISM, electrocatalysis has also shown significant potential for application in mineral ion monitoring in plants. Ibrahim et al. [76] developed a plant nitrate sensor based on a photosensitive epoxy resin, consisting of photoresist (SU8), an artificial enzyme containing Co^2+^/Co^3+^, and graphene oxide, where Co ions possess electrocatalytic activity. The sensor showed a detection range of 0.2–2000 ppm with a detection limit as low as 0.2 ppm and a long shelf life, making it suitable for long-term in situ monitoring of nitrate ions in plants (Figure 7b).

#### 3.1.3. Organic Substance Detection

Phytohormones, such as indole-3-acetic acid (IAA), salicylic acid (SA), gibberellin (GA), abscisic acid (ABA), and ethylene, are trace amounts of organic chemicals in plants that are essential for the regulation of plant growth, flowering, fruit ripening, and the response to environmental adversity. For example, SA enhances plant stress tolerance by regulating plant stomata and transpiration; IAA is involved in plant response to salt stress; and ABA plays an important role in plant organ dormancy, maturation, and senescence.

**Figure 8:**
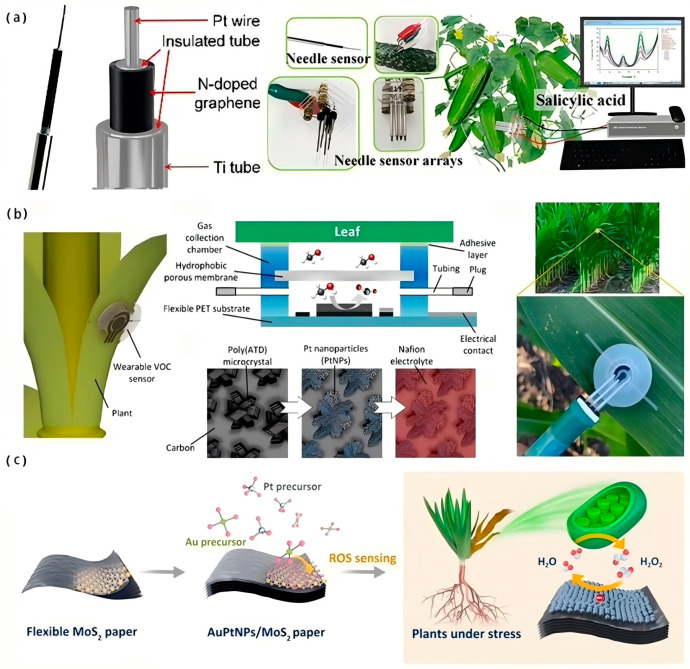
Example of organic substance detection application. (**a**) Synthesized self-supporting nitrogen-doped graphene microelectrodes based on a three-electrode system for the detection of SA in cucumber stems [77]. Copyright 2021, Elsevier. (**b**) A low-cost, portable, and wearable electrochemical sensor based on the principle of electrocatalytic VOC-methanol redox [12]. Copyright 2022, ACS. (**c**) A molybdenum disulfide paper-based electrochemical sensor based on noble metal alloyed nanoparticles (AuPtNPs) modification for monitoring ROS in plants [80]. Copyright 2020, Elsevier.

In recent years, needle-like three-electrode electrochemical sensors have attracted attention because of their high sensitivity in the detection of phytohormones. For example, Liu et al. [9] constructed an Au@Cu_2_O-Gr-PDA sensing layer on an Al microelectrode to build a three-electrode electrochemical sensor and successfully realized high-precision real-time monitoring of SA concentration in cucumber. Similarly, to enhance the electrocatalytic interaction between the sensing layer materials, Wang et al. [10] adopted the same strategy to develop composite nanoparticles of graphene, Au, and SnO_2_ as sensing electrodes and verified the feasibility of quantitative ABA monitoring in cucumber. Zhang et al. [77] synthesized self-supporting nitrogen-doped graphene microelectrodes based on a three-electrode system for the detection of SA in cucumber stems (Figure 8a). However, they observed severe electrode contamination caused by SA films on the electrode surface. To solve the problem of electrode inactivation by contamination, Li [78] et al. developed a disposable and low-cost IAA electrochemical microsensor using SS filament electrodes for detecting IAA in soybean seedlings under salt stress.

Plants also transmit information and adapt to the environment by releasing volatile organic compounds (VOCs). Wearable plant sensors can realize real-time monitoring of plant physiological conditions as well as disease prevention through tracking the changes in VOC. Li et al. [11] developed a smartphone-integrated plant VOC analysis platform that can identify ten common plant volatiles in less than one minute for accurate early detection of late blight in tomato leaves. Note that the sensor may drift in certain toxic gas environments, suggesting that the use of the VOC analysis platform may be limited in certain special cases, such as proximity to decaying vegetables or fruits. On this basis, Lee et al. [79] developed a multifunctional wearable sensor that enables simultaneous measurement of VOCs, leaf surface temperature/humidity, and ambient humidity. The sensor not only enables early detection of tomato pathogens but also tracks plant water loss. In addition, the team combined machine learning data analysis with the sensors to enable quantitative detection of tomato spotted wilt virus up to 4 days after inoculation and to predict the optimal sensor combination. This research brings new inspiration and enlightenment in plant disease detection, stress phenotyping, and predictive analysis. Based on the principle of electrocatalytic VOC-methanol redox, a low-cost, portable, and wearable electrochemical sensor was developed by Ibrahim et al. [12]. The sensor used PtNPs-modified poly(ATD) as a catalytic material and achieved detection of methanol emissions at the sub-ppm level with a response time of <20 s (Figure 8b). Although wearable devices for plant VOC monitoring are promising, the stability and selectivity in dealing with different stressors and pathogenic bacterial infections need to be further investigated.

Reactive oxygen species (ROS) are by-products of plant aerobic metabolism and are involved in cellular signaling and the plant response to adversity. Yao et al. [80] prepared a molybdenum disulfide paper-based electrochemical sensor based on noble metal alloyed nanoparticle (AuPt NP) modification for monitoring ROS in plants (Figure 8c). Among all the ROS molecules, H_2_O_2_ has the longest half-life (1 ms) and can be used as an important indicator for monitoring plant adversity stress as well as health status. Based on this principle, Sun et al. [6] developed a paper-based analyzer consisting of disposable nanogold-modified indium tin oxide (ITO) working electrodes for monitoring H_2_O_2_ concentration in tomato leaves infected with gray mold over 24 h. It is important to note that the tomato leaf sampling using a 4 mm diameter Miltex biopsy punch during the study was invasive and destructive, which may cause tomato tissue damage and senescence.

#### 3.1.4. Electrical Signal Detection

In addition to hormone regulation, the electrical pathway is regarded as another key signaling pathway in plants. As an important physiological signal involved in physiological regulation and information transmission in plants, electric signals are usually accompanied by their generation in plant growth and development as well as various physiological activities. Especially under adverse conditions, the transmission of electric signals is faster than the symptoms that appear on the surface [81]. Therefore, monitoring changes in plant electrical signals can reflect the physiological information and health status of plants.

**Figure 9:**
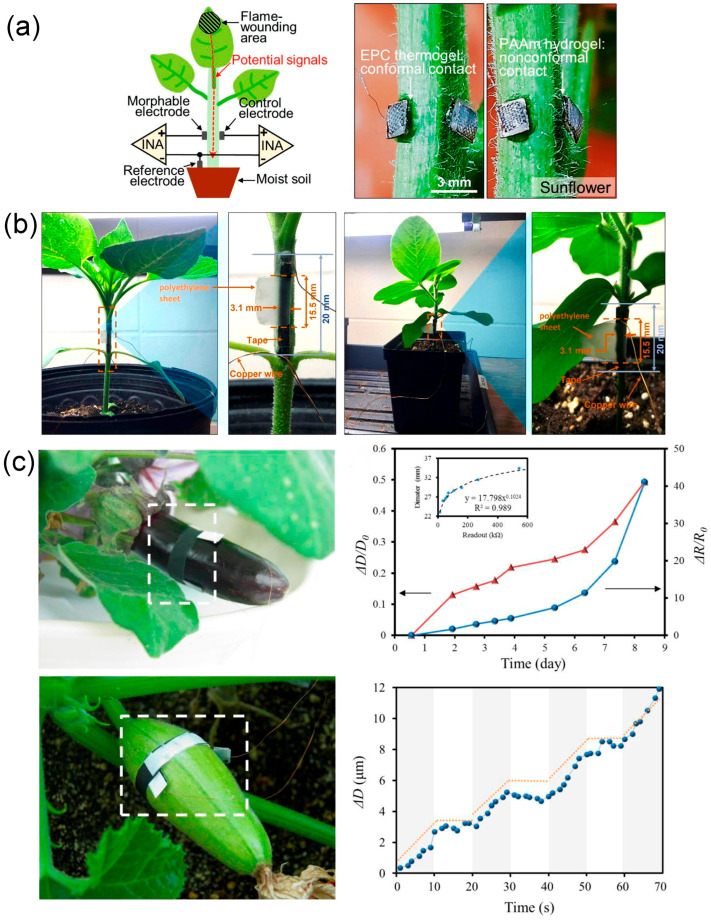
Example of electrical signal detection and plant growth detection application. (**a**) A deformable ion electrode made of thermogel, which was securely locked to an irregular, hairy surface. A metal plate was placed on top of the thermal electrode, thus successfully establishing a self-adhesive, conformal, and mechanically strong electrical interface on the hairy plant surface, realizing the recording of high-fidelity electrophysiological signals [82]. Copyright 2021, Wiley. (**b**) A polyaniline (PANI)/elastic tape-based strain sensor, and by attaching the sensor to the stem internodes of sunflower and soybean, growth monitoring at the early stages of sunflower and soybean plants was realized [85]. Copyright 2023, Wiley. (**c**) Attach sensors to the *Solanum melongena* L. (the white dotted frame )to quantitatively collect growth signals and output resistance signals [86]. The second graph shows wearable device (●) and manual measurements (▲) for growth measurements over a 9 day period. And the fourth graph demonstrates that the diameter of the Cucurbita pepo exhibits a rhythmic growth pattern (illustrated with dash lines) as it grows in seconds. Copyright 2019, Elsevier.

Luo et al. [82] used a deformable ion electrode of thermogel, which was securely locked to an irregular, hairy surface. A metal plate was placed on top of the thermal electrode, thus successfully establishing a self-adhesive, conformal, and mechanically strong electrical interface on the hairy plant surface, realizing the recording of high-fidelity electrophysiological signals. Eventually, the fluctuation of electrical signals generated when the plant was subjected to abiotic stresses, such as fire and mechanical damage, was successfully detected (Figure 9a). Although the flexible sensor successfully achieved interfacial bonding with plants with irregular and hairy surfaces, the high concentration of ion-conductive gel used to enhance the adhesion and conductivity of the electrodes caused a certain degree of damage to the plants. It is known that the epidermal structures of plants vary, which means that the flexible electrodes adhered to the plant surface should also be different. In response to the smooth nature of the cactus epidermis, Ochiai et al. [83] proposed a plant monitoring system based on boron-doped diamond (BDD) electrodes. The sensor transducer was connected to the bast tissue of the plant and monitored the biopotential inside the plant. The BDD sensor detected significant changes in the biopotential when a finger touched the hybridized surface of the cactus or when environmental factors such as temperature and humidity changed. Note that the BDD electrode is 4–7 times more sensitive to changes in the biopotential of potted cactus hybrid plants compared to Pt or Ag electrodes, and it can be used as a monitoring system for plant growth and as an early warning system for environmental events to monitor changes in plant biopotential effectively for a long period.

#### 3.1.5. Plant Growth Detection

Through real-time monitoring of plant growth, we can grasp the growth status of plants promptly and accordingly optimize planting strategies to maximize crop yields. Plant growth is a highly dynamic process that is not always fixed, making the selection of appropriate measurement tools particularly important [84]. Currently, there are several methods for measuring plant growth, for example, the visual inspection image processing technique. This technique extracts the image information of the measured object and is processed by an algorithm to realize nondestructive monitoring of plant growth. However, the monitoring results are easily influenced by the environmental lighting conditions, and for some complex plant growth patterns, the image recognition and algorithm processing may produce obvious errors. Along with the continuous development of wearable sensor devices, researchers have tried to design wearable devices that can be directly attached to plant stalks for real-time monitoring of plant growth. The wearable technology can be better adapted to plant growth changes and thus provide more accurate and stable monitoring data.

Nassar et al. [13] prepared a stretchable strain sensor to monitor the growth of barley stalks. A thin layer of gold film was deposited on the PDMS as a strain-sensing material, and the gold film cracked when subjected to the strain, causing a change in resistance. The strain sensor was attached to the stalk of barley, and the growth rate was calculated by detecting the change in resistance of the strain sensor, realizing micrometer-scale monitoring of barley growth. Similarly, Borode et al. [85] developed a polyaniline (PANI)/elastic tape-based strain sensor, and by attaching the sensor to the stem internodes of sunflower and soybean, growth monitoring at the early stages of sunflower and soybean plants was realized (Figure 9b). The sensor successfully monitored the micrometer-scale growth of plant stalks within 24 h, and the recorded growth rates and patterns of sunflower and soybean stalks were highly consistent with existing plant growth datasets. This low-cost sensor shows great potential for application in plant growth monitoring. However, it may have performance limitations in field environments with large temperature fluctuations.

In addition to monitoring the plant stem growth, a stretchable strain sensor can also be used to detect the fruit expansion process and leaf growth condition. TANG et al. [86] successfully prepared a stretchable strain sensor based on graphite and CNTs, which realized the dynamic measurement of plant growth on the nanoscale. The synergistic enhancement effect of graphite and carbon nanotubes provides a method for the rapid fabrication of high-performance wearable strain sensors. The sensor was fixed on *Solanum melongena* L. to quantitatively collect growth information and output resistance signals, which were converted by the readout circuit to obtain intuitive and readable results to accurately deduce the *Solanum melongena* L. growth pattern (Figure 9c). The integration of the sensor and the readout circuit opens up a promising new avenue for plant growth measurement. Similarly, the multifunctional sensor, single-walled carbon nanotubes (SWCNTs), as proposed by Zhao et al. [14], can be used to monitor not only the microclimate information around the plant, such as temperature and light, but also the growth of plant leaves.

Although the above sensors can detect and monitor the growth of the plant itself (such as stems and leaves) or plant fruits, the detection range of plant growth is limited due to the material itself and the structural design. Wang et al. [87] introduced a highly flexible, robust, and wireless conductive polymer-based strain sensor. The conductive polymer has a higher stretchability compared to other fabricated materials and exhibits an unprecedented operating strain of over 400% in monitoring Avena sativa growth, minimizing any hindrance to the natural growth pattern of the plant. To minimize environmental disturbances such as RH, temperature, and light, the sensor was encapsulated using styrene–ethylene–butene–styrene (SEBS). Additionally, the study paid attention to the high light transparency (98.7%) and low weight (45 mg) of the wearable sensor to minimize interference with plant growth.

### 3.2. Environmental Information Detection

**Figure 10:**
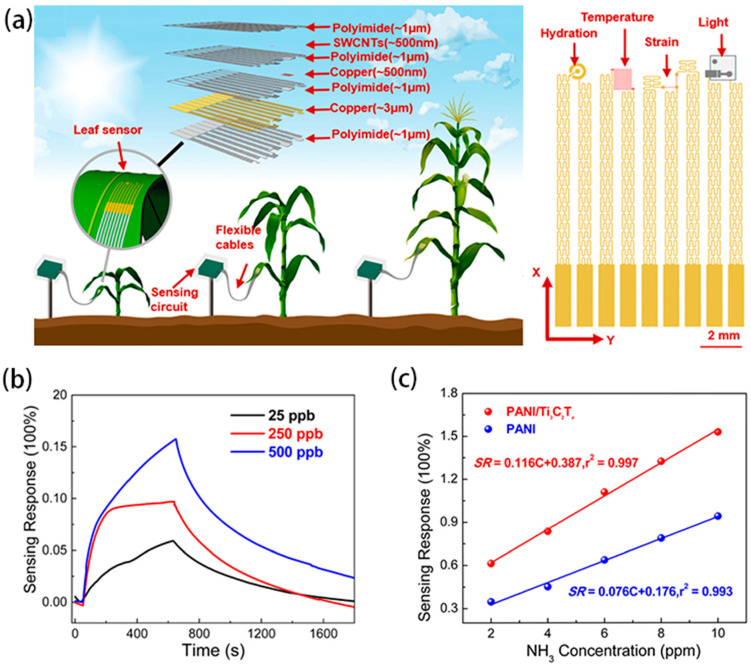
Example of environmental information detection application. (**a**) Schematic diagram and exploded view of a leaf sensor [14]. Copyright 2019, ACS. (**b**) The real-time response–recovery curves of NH_3_ sensors based on PANI/Ti_3_C_2_T_x_ hybrid sensitive films to 25–500 ppb NH_3_ at 20 °C and under dry air [15]. Copyright 2020, Elsevier. (**c**) Curves of the sensing response as a function of NH_3_ concentration for two types of sensitive films for NH_3_ sensors based on pure PANI and PANI/Ti_3_C_2_T*_x_* hybrid sensitive films for NH_3_ concentrations ranging from 2.0 to 10.0 ppm [15]. Copyright 2020, Elsevier.

The growth and development processes of plants are largely affected by external environmental factors such as light, temperature, humidity, and gas/pesticide residues. Clarifying the interaction between plants and their microenvironment can ensure a favorable environment for plants and enhance the yield of crops. Wearable sensors for plants have emerged that can detect multiple environmental factors in one sensor device. For instance, Nassar et al. [13] developed a wearable device for plants with integrated temperature, humidity, and strain sensors. The device is integrated on top of a flexible and ultra-lightweight butterfly-shaped polyimide (PI)/PDMS platform, which can be tightly placed on the soft surface of any plant to monitor the growth of the plant along with the detection of environmental conditions. Similarly, Zhao et al. [14] constructed a multifunctional and extensible foliar sensor. To ensure that the sensor can closely fit the foliage surface and monitor the strain signals induced by leaf growth as well as the foliar microenvironmental information such as temperature, light intensity, and humidity, the ultra-thin elastic substrate PDMS was used to reduce the tensile constraint of the sensor, and an island–bridge style with a serpentine structure was designed (Figure 10a). The entire fabrication process of the sensor is relatively complicated and requires the use of multiple passes of magnetron sputtering and oxygen plasma etching to achieve its multifunctionality. To address these issues, Di Tocco et al. [88] provide a simple, cost-effective solution to manufacture strain sensors based on plant size requirements and integrate them into a multi-sensor platform for continuous monitoring of the microclimate and growth. In addition, they encapsulated the sensors used for growth monitoring into a silicone matrix to help dampen the effects of climate variability on sensor output. Sensor readings in general are susceptible to environmental variations, and encapsulating the sensing sensor in polymer is a potential solution.

In addition, toxic gases in the environment such as NH_3_, CO_2_, and O_3_ can cause irreversible damage to plants. Li et al. [15] designed a flexible gas sensor based on a hybrid (PANI)/Ti_3_C_2_T*_x_*-sensitive membrane aimed at monitoring NH_3_ around plants (Figure 10b,c). The sensor exhibits significant advantages over pure PANI sensors. In the typical concentration range (2–10 ppm) corresponding to real agricultural ammonia volatilization, the sensor’s response to NH_3_ concentration value is at concentrations of 0.387 ppm^−1^, which is significantly higher than that of the pure PANI-sensitive film, which is 0.176 ppm^−1^. At the same time, it can detect ammonia concentrations as low as 25 ppb, significantly broadening the concentration range of PANI films for NH_3_ detection. In particular, the sensing performance of the sensor air with 20–80% RH and with a temperature of 10–40 °C showed no obvious degradation, making it promising for practical agricultural applications. Li et al. [16] prepared a gas sensor array to detect NO_2_ around plant leaves using metallic single-walled carbon nanotubes as conductive electrodes and silver nanoparticle-modified reduced graphene oxide (AgNPs/rGO) as the sensing layer. The sensor exhibited a sensitive response to 0.2–20 ppm NO_2_, mechanical robustness (3000 bending cycles), and a significant sensing capability for NO_2_ down to 0.2 ppm at room temperature. Notably, the preparation method shows good scalability and applicability on planar and non-planar supports, which bodes well for its great potential in practical applications. This work provides a reliable platform for the study of flexible devices for multifunctional sensing applications.

In addition, microenvironmental information such as plant pests, pesticide residues, and diseases can also be detected by wearable plant sensors. Mishrai et al. [89] reported a glove-integrated flexible sensor that can rapidly monitor organophosphorus (OP) neurotoxicant compounds on crop surfaces. An organophosphorus hydrolase (OPH)-based biosensor system on the index finger is capable of rapid on-site detection of OP compounds on suspected surfaces and agricultural products after sliding the thumb to collect samples, achieving successful integration of OP analysis into the user’s fingertips. This portable and efficient detection technology not only provides valuable ideas for the design of next-generation plant flexible sensors but is also expected to drive researchers to further develop more integrated and miniaturized plant flexible sensors.

## 4. Challenges and Prospects

**Figure 11:**
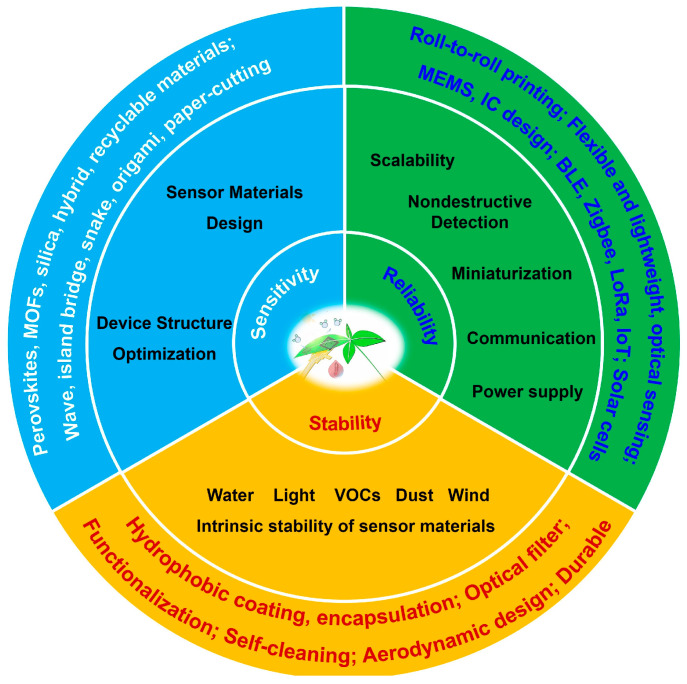
The challenges of wearable plant sensors.

In comparison to traditional rigid sensors, miniaturized, flexible, and extensible plant sensors are more effective for real-time, in situ, long-term monitoring of plants. Currently, most of the plant wearable sensors are still at an experimental stage, and several substantial challenges must be addressed before their practical applications in outdoor agriculture. As presented in Figure 11, these challenges include improving the sensor sensitivity performance and ensuring the sensor reliability and stability in complex farm environments. Further improvements and enhancements across multiple domains are essential to overcome these obstacles.

To improve the sensor sensitivity, we should carefully design the sensor materials and device structure. The choice of materials significantly impacts the sensor’s ability to detect small, subtle changes in plant parameters. Materials like conductive polymers, elastomers, hydrogels (e.g., polypyrrole, polyaniline, PEDOT:PSS, and polydimethylsiloxane), metal nanoparticles (e.g., silver and gold), graphene and its derivatives, carbon nanotubes (CNTs), and metal oxides (e.g., zinc oxide and tin oxide) are frequently employed for their excellent electrical conductivity, flexibility, and responsiveness to environmental stimuli. These materials enable the detection of a wide range of plant parameters, such as water stress, transpiration rates, and metabolic activities like photosynthesis or respiration. Some of the above-mentioned materials are expensive or unstable, and some of them need high working temperatures to ensure high responsivity. To further enhance the performance of these sensors, we can develop low-cost and stable sensor materials that can work at room temperature. These emerging and promising materials include inorganic lead-free perovskites, mesoporous metal–organic frameworks (MOFs), silica nanoparticles, organic/inorganic hybrid materials, biodegradable or recyclable materials, and so on. Additionally, the structural designs of the electrode or sensor materials such as using wave structures, island–bridge structures, self-similar snake structures, origami structures, and paper-cutting structures are also promising to achieve high-performance sensors.

To ensure the reliability of wearable plant sensors, significant advancements are required in sensor scalability, nondestructive detection, miniaturization, wireless communication, and power supply. Scalability can be improved through the implementation of standardized manufacturing processes, such as roll-to-roll printing, and the utilization of cost-efficient, durable materials that can be easily replicated and adapted to different plant types or environmental conditions. Nondestructive detection can be achieved by employing flexible and lightweight materials, such as silicone, graphene, or conductive polymers, in combination with noninvasive attachment mechanisms or optical sensing technologies, such as fluorescence imaging, to minimize the physical impact on plant structures. The miniaturization of sensors can produce small, reliable, highly sensitive sensors capable of integrating multiple functionalities without impeding plant growth. Miniaturization can be facilitated through the use of high-performance nanomaterials as discussed above, microfabrication techniques such as microelectromechanical systems (MEMS), and integrated circuit design. Wireless communication can be augmented by adopting low-power communication protocols, such as Bluetooth Low Energy (BLE), Zigbee, or LoRa, designing robust antenna systems for stable data transmission, and integrating sensors with Internet of Things (IoT) platforms for real-time monitoring, with redundancy mechanisms to mitigate potential signal interference. Ensuring a reliable and sustainable power supply involves the incorporation of multiple energy harvesting technologies such as solar cell panels, thermoelectric generators and vibration-based energy harvesters, long-lasting batteries that can withstand prolonged use and extreme conditions, dynamic power management systems, and wireless energy transfer methods that can recharge sensors without direct contact. Collectively, these innovations are critical for the development of accurate and reliable wearable sensors suitable for diverse agricultural applications.

To enhance the stability of wearable plant sensors in complex agricultural environments, it is imperative to address the challenges posed by water, light, complex VOCs, dust, wind, and the intrinsic stability of sensor materials. Water resistance can be achieved through the application of hydrophobic or waterproof coatings on sensor components, along with encapsulating sensitive electronics in moisture-resistant materials such as silicone or polyimide. Regarding light exposure, UV-stabilized materials and optical filters can be incorporated to prevent photodegradation or interference from excessive solar radiation. Addressing volatile organic compounds requires the use of chemically selective materials, such as functionalized carbon nanotubes or metal–organic frameworks (MOFs), to ensure accurate measurements amidst fluctuating VOC concentrations. For dust protection, robust sensor enclosures and surface treatments designed to repel particulates can enhance stability, while self-cleaning nanostructured coatings reduce maintenance requirements. To counteract the effects of wind, aerodynamic sensor designs, flexible materials capable of withstanding mechanical stress, and vibration-dampening mechanisms are necessary to maintain data accuracy in turbulent conditions. Moreover, improving the stability of sensor materials is critical, which involves selecting durable, environmentally resilient materials that can resist degradation due to chemical exposure, temperature fluctuations, and long-term field deployment. These integrated strategies ensure the high stability of wearable plant sensors in diverse and demanding agricultural environments.

To summarize, the advances in flexible electronics and materials science will enable compact, lightweight, and noninvasive sensor devices that seamlessly integrate with plants, offering multi-modal detection for real-time monitoring of environmental information and plant physiological information for predictive analytics. By integrating cutting-edge technologies such as IoT and AI, along with sustainable designs, wearable plant sensors will enhance agricultural productivity and sustainability. Practical long-term field use will be realized in the future when high-performance, reliable, and stable wearable sensors are available. Together with scientists and experts in the field, we are confident that wearable plant sensors will continue to achieve significant advancements and play a vital role in the development of smart agriculture.

## Figures and Tables

**Table 1:** Comparison of the performance of pressure sensors with different sensing mechanisms.

Sensing Mechanism	Advantages	Limitations
Piezoresistive	Simple sensing mechanism Low power consumption Strong anti-interference Easy to collect and verify data	Large signal drift
Capacitive	Simple structure High sensitivity Low energy consumption Small signal drift	Poor environmental stability Limited compressibility of the dielectric layer
Piezoelectric	Suitable for dynamic force measurement Low power consumption Self-powered Short response time	Not capable of static force measurement Susceptible to temperature

**Table 2:** Performance comparison of various types of flexible temperature sensors.

Type	Advantages	Limitations
Flexible resistance temperature detectors	Good linearity Wide working range Good stability	Long response time High cost High power supply
Flexible thermocouples	Low cost Short response time Simple structure	Low accuracy Poor stability Poor sensitivity
Flexible thermistors	Strong response Low cost Small scale Short response time	Nonlinear Narrow operating range High power supply
Flexible thermochromic	Visualization Short response time	Poor stability Narrow working range poor accuracy

## Data Availability

The data are available from the corresponding author on reasonable request.
